# iEnhancer-DCLA: using the original sequence to identify enhancers and their strength based on a deep learning framework

**DOI:** 10.1186/s12859-022-05033-x

**Published:** 2022-11-14

**Authors:** Meng Liao, Jian-ping Zhao, Jing Tian, Chun-Hou Zheng

**Affiliations:** 1 College of Mathematics and System Sciences, Xinjiang University, Ürümqi, China; 2 School of Computer Science and Technology, Anhui University, Hefei, China

**Keywords:** Enhancer, Word embedding, k-mers, Convolutional neural network, Bidirectional long short-term memory network, Attention mechanism

## Abstract

Enhancers are small regions of DNA that bind to proteins, which enhance the transcription of genes. The enhancer may be located upstream or downstream of the gene. It is not necessarily close to the gene to be acted on, because the entanglement structure of chromatin allows the positions far apart in the sequence to have the opportunity to contact each other. Therefore, identifying enhancers and their strength is a complex and challenging task. In this article, a new prediction method based on deep learning is proposed to identify enhancers and enhancer strength, called iEnhancer-DCLA. Firstly, we use word2vec to convert k-mers into number vectors to construct an input matrix. Secondly, we use convolutional neural network and bidirectional long short-term memory network to extract sequence features, and finally use the attention mechanism to extract relatively important features. In the task of predicting enhancers and their strengths, this method has improved to a certain extent in most evaluation indexes. In summary, we believe that this method provides new ideas in the analysis of enhancers.

**Supplementary Information:**

The online version contains supplementary material available at 10.1186/s12859-022-05033-x.

## Introduction

Gene enhancers are non-coding segments of DNA that play a central role in regulating transcriptional processes that control development, cell identity, and evolution [1]. Recently, a large number of enhancers of humans and other species (both eukaryotes and prokaryotes) have been recognized [2]. The number of enhancers in mammals ranges from 50,000 to 100,000. Most enhancers are located in intron region and intergenic region, and a few are located in exon region [3]. The enhancer contains a variety of genetic marker sites, the most common is the transcription factor binding site. Enhancers regulate gene expression by interacting with their target gene promoters. This interaction may be in cis or in trans. Cis action refers to the enhancer and its action site genes on the same chromosome, while trans action refers to the enhancer and its action site genes on different chromosomes [4]. On average, each promoter interacts with 4.9 enhancers [5]. Super-enhancers (SEs) are large clusters of transcriptionally active enhancers, often located near cell-specific functional genes. Although super enhancers have been widely used in many studies, there is no clear definition [6]. In addition, many human diseases have been shown to be affected by genetic variations in enhancers [7], such as various cancers [8] and inflammatory bowel disease [9]. Therefore, the identification of enhancers and the prediction of their action sites have always been a hot topic in related fields.

One of the basic problems of enhancer research is enhancer prediction. In order to find the properties and functions of enhancers, it is necessary to identify the locations of enhancers on the genome. For a long time in the past, the prediction of enhancers has relied on biological experimental techniques. For example, Conservative analysis was performed using sequence conserved data and transcription factor binding site data to predict enhancers. [10–12]. And using DNase I hypersensitivity sites sequencing data to identify enhancers based on chromatin accessibility [13]. However, these methods result in a high false-positive rate because the data contain sequences of other regulatory elements that are not enhancers binding to transcription factors. In addition, the method of predicting enhancers using ChIP-seq data of transcription factors and ChIP-seq data of transcription coactivator P300 has been widely used [14–16], but it is not effective to distinguish strong enhancers from weak enhancers. Prediction based on eRNA data is another approach [17–19]. The enhancer transcribes eRNA, which is detected by sequencing technology and mapped back to the original genome to obtain the location information of the enhancer. The disadvantages are that a large sample size is required, and all methods for determining the location of enhancers based on eRNA data cannot be used to predict unexpressed enhancers.

Biological experiments are time-consuming and costly. With the rapid development of machine learning and deep learning, many prediction models have been built to identify enhancers and their strength. iEnhancer-2 L is the first predictive model that can identify not only intensifiers but also their strength [20]. iEnhancer − 2 L uses pseudo k-tuple nucleotide composition (PseKNC) as the encoding method of sequence characteristics. EhancerPred uses bi-Bayes and pseudo-nucleotide composition as feature extraction method [21]. iEnhancer-EL is an upgraded version of iEnhancer-2 L [22]. Its two stages consist of 16 key individual classifiers, all of which are selected from 171 basic classifiers formed based on subsequence profile, kmer and PseKNC. The above three machine learning models are based on support vector machines (SVM) to construct classifiers. iEnhancer -ECNN uses one-hot encoding and k-mers to process the data, and uses CNN to construct the ensemble model [23]. But one-hot encoding is vulnerable to the problem of dimensionality disaster and ignores the correlation information between k-mer words. iEnhancer -XG combines five features (k-spectrum profile, mismatch k-tuple, subsequence profile, position-specific scoring matrix) and constructs a two-layer predictor using “XGBoost” as the basic classifier [24]. iEnhancer-EBLSTM uses 3-mer to encode the input DNA sequences and then predicts enhancers by bidirectional LSTM [25]. These methods can identify and classify enhancers and their strength. But the accuracy of layers 1 and 2 predictors needs to be improved further, and it should be possible to develop better models using the new deep learning framework.

In this study, we propose a new deep learning prediction framework called iEnhancer-DCLA. In the first stage of the model, enhancers are identified. In the second stage, we classified enhancers’ strength. The main idea of the model is to combine word embedding and k-mers to encode sequence data, and then use CNN, Bi-LSTM and attention mechanism to extract features and classify them. Meanwhile, we use SHapley Additive explanation [26] algorithm to explain the influence of the features extracted from the model. The experimental results in the independent test dataset show that this method has better performance than some existing methods. The source codes and data are freely at https://github.com/WamesM/iEnhancer-DCLA.

## Materials and methods

### Benchmark dataset

The benchmark dataset used in this article is divided into two parts: the training dataset and the independent test dataset. The dataset used in our experiment was obtained from the study of Liu et al. [20]. In order to facilitate a fair comparison with previous studies, this dataset has also been used to classify enhancers in later studies, such as in the development of EnhancerPred [21], iEnhancer-EL [22], iEnhancer- ECNN [23], and iEnhancer- XG [24]. In this dataset, enhancer sequences of 9 different cell lines were collected, from which a 200 bp fragment of the same length was extracted. The CDHIT [27] software was then used to exclude paired sequences with sequence similarity greater than 20%. The training dataset included 1484 enhancer sequence samples (742 strong enhancers and 742 weak enhancers) and 1484 non-enhancer sequence samples. To evaluate the generalization performance of our model, the independent test dataset is set up. The independent test dataset includes 200 enhancer sequence samples (100 strong enhancers and 100 weak enhancers) and 200 non-enhancer sequence samples.

### Sequence representation

In many deep learning algorithms for processing biological sequences, the method of using natural language processing technology to extract features from the original DNA sequence is widely used [28–30]. K-mer analysis is an effective method in DNA sequence analysis. K-mer splits a sequence into substrings of k bases. When the step size is 1, the DNA sequence with length l is divided into (l − k + 1) k-mers. For example, when we set k = 7, the sequence ‘ACGTCGACG’ is split into three 7-mers: ‘ACGTCGA’, ‘CGTCGAC’, and ‘GTCGACG’. This makes the sequence easier to calculate and understand. We treat the entire DNA sequence as a sentence, and the k-mer fragments as words. We derive the distributed representation matrix by connecting the dna2vec [31] method. Dna2vec is based on the popular word embedding model word2vec [32]. In our model, dna2vec was pretrained with hg38 human components chr1 to chr22, and then adapted to our predictive task using our datasets. Finally, each k-mer word is represented as a 100-dimensional vector. In this experiment, we set k to 7 and converted each 200 bp enhancer sequences into a (194,100) matrix.

### Model architecture

**Fig. 1:**
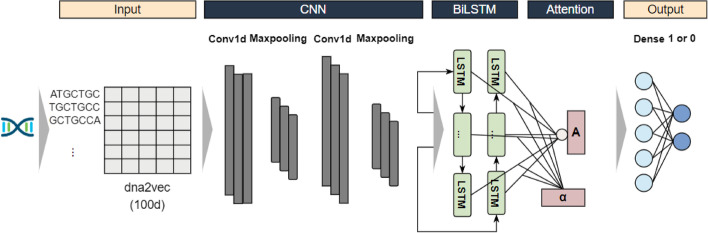
Model structure. It includes feature representation based on dna2vec method, two convolutional layers, two pooling layers, bidirectional long short-term memory network layer, attention layer and finally two fully connected layers

We propose a two-stage deep learning prediction model using DNA sequences of enhancers for classification. The first stage is to identify enhancers. The second stage is to identify the strength of enhancers. In fact, the first stage has the same network structure as the second stage. The only difference between the two stages is the dataset used. During the training in the first stage, all data are used as training dataset and are classified as enhancers and non-enhancers. In the second stage, only the enhancers are used in the experiment and are classified as strong enhancers and weak enhancers. The workflow of the model is shown in Fig. 1. The model consists of five modules, including sequence words embedding input, convolutional neural network extracting sequence features, bidirectional long short-term memory network extracting sequence long-term dependence information, attention mechanism extracting relatively more important features, and predicting output.

#### Convolutional neural network (CNN)

CNN is a kind of Feedforward Neural Networks with deep structure and convolution computation, which is one of the representative algorithms of deep learning [33, 34]. Our convolution module consists of one-dimensional convolution layer, rectified linear layer (ReLU) [35], batch normalization layer and max pooling layer. In order to avoid overfitting, a dropout layer [36] with a dropout rate of 0.2 was used in the middle. In the first convolutional layer, the number of convolutional kernels is set to 256, the size of the convolutional kernels is set to 8, the stride is set to 1, and the length of max pooling layer is set to 2. In the second convolutional layer, the number of convolutional kernels is set to 128, the size of the convolutional kernels is set to 8, the stride is set to 1, and the length of max pooling layer is set to 2.

#### Bidirectional long short-term memory network (LSTM)

LSTM is a special type of recurrent neural network that can learn long-term dependency information. On many issues, LSTM has achieved great success and been widely used, such as DeepD2V [37]. Because DNA sequences are double-stranded, we use Bi-LSTM to capture the long-term dependence of the sequence. Bi-LSTM layer is composed of forward and reverse parts to learn features. The calculation formula is as follows:

1 $${f_t}=\sigma \left( {{W_f}{x_t}+{U_f}{h_{t - 1}}+{b_f}} \right)$$

2 $${i_t}=\sigma \left( {{W_i}{x_t}+{U_i}{h_{t - 1}}+{b_i}} \right)$$

3 $$\widetilde {{{C_t}}}=\tanh \left( {{W_C}{x_t}+{U_C}{h_{t - 1}}+{b_C}} \right)$$

4 $${C_t}={f_t} \odot {C_{t - 1}}+{i_t} \odot \widetilde {{{C_t}}}$$

5 $${o_t}=\sigma \left( {{W_o}{x_t}+{U_o}{h_{t - 1}}+{b_o}} \right)$$

6 $${h_t}={o_t} \odot \tanh \left( {{C_t}} \right)$$

The Eq. (1) represents the forgetting gate to decide which information should be discarded or retained. The Eqs. (2) and (3) represent the input gate, which is used to decide which information to update and create a new candidate value vector. The Eq. (4) is used to calculate the current cell state. The Eq. (5) represents the output gate, which is used to calculate the value of the next hidden state. Where ${W}_{f}$, ${W}_{i}$, ${W}_{C}$, ${W}_{o}$, ${U}_{f}$, ${U}_{i}$, ${U}_{C}$, ${U}_{o}$ are weights, and ${b}_{f}$, ${b}_{i}$, ${b}_{C}$, and ${b}_{o}$ are biases. We set the number of neurons into the Bi-LSTM layer to 64.

#### Attention

In the field of Artificial Intelligence (AI), attention mechanism has become an important part of neural network structure. It has a large number of applications in natural language processing, statistical learning, speech and computer [38, 39]. The core of the attention mechanism is to introduce attention weight to the features learned in the previous layer and assign different weight to each feature to learn the relatively more important features.

7 $${u_i}=\tanh \left( {{W_s}{h_i}+{b_s}} \right)$$

8 $${\alpha _i}=\frac{{\exp \left( {u_{i}^{T}{u_s}} \right)}}{{\sum\nolimits_{{i=1}}^{L} {\exp \left( {u_{i}^{T}{u_s}} \right)} }}$$

9 $$A=\sum\nolimits_{{i=1}}^{L} {{\alpha _i} * {h_i}}$$

where ${W}_{s}$, ${b}_{s}$ and ${u}_{s}$ are the variables that need to be learned; ${\alpha }_{i}$obtained through calculation represents the importance of ${h}_{i}$; ${h}_{i}$ is the output of bi-LSTM layer at the i time; A represents the feature vector after finally passing through the attention mechanism layer. We set up 64 output units in the attention layer.

Finally, the model is connected to two fully connected layers for prediction, and the sigmoid activation function is used to calculate the probability of classification into a certain category. Dimension changes of iEnhaner-DCLA under each module in Additional file 1: Fig. S1.

### Evaluation parameters

In order to evaluate the performance of the model objectively and comprehensively, we use the following metrics to evaluate the predictive performance of the model: (1) Accuracy (ACC), (2) Sensitivity (Sn), (3) Specificity (Sp), (4) Matthews correlation coefficient (MCC), (5) Area Under the ROC Curve (AUC), (6) Area Under the Precision Recall Curve (AUPR), (7) F1-score. The formula of evaluation index is as follows:

10 $$ACC=\frac{{TP+TN}}{{TP+TN+FP+FN}}$$

11 $$Sn=\frac{{TP}}{{TP+FN}}$$

12 $$Sp=\frac{{TN}}{{TN+FP}}$$

13 $$MCC=\frac{{TP \times TN - FP \times FN}}{{\sqrt {\left( {TP+FP} \right)\left( {TP+FN} \right)\left( {TN+FP} \right)+\left( {TN+FN} \right)} }}$$

where TP, FP, TN and FN represent true positive, false positive, true negative and false negative values respectively.

## Results and discussions

### Analysis of DNA sequences

**Fig. 2:**
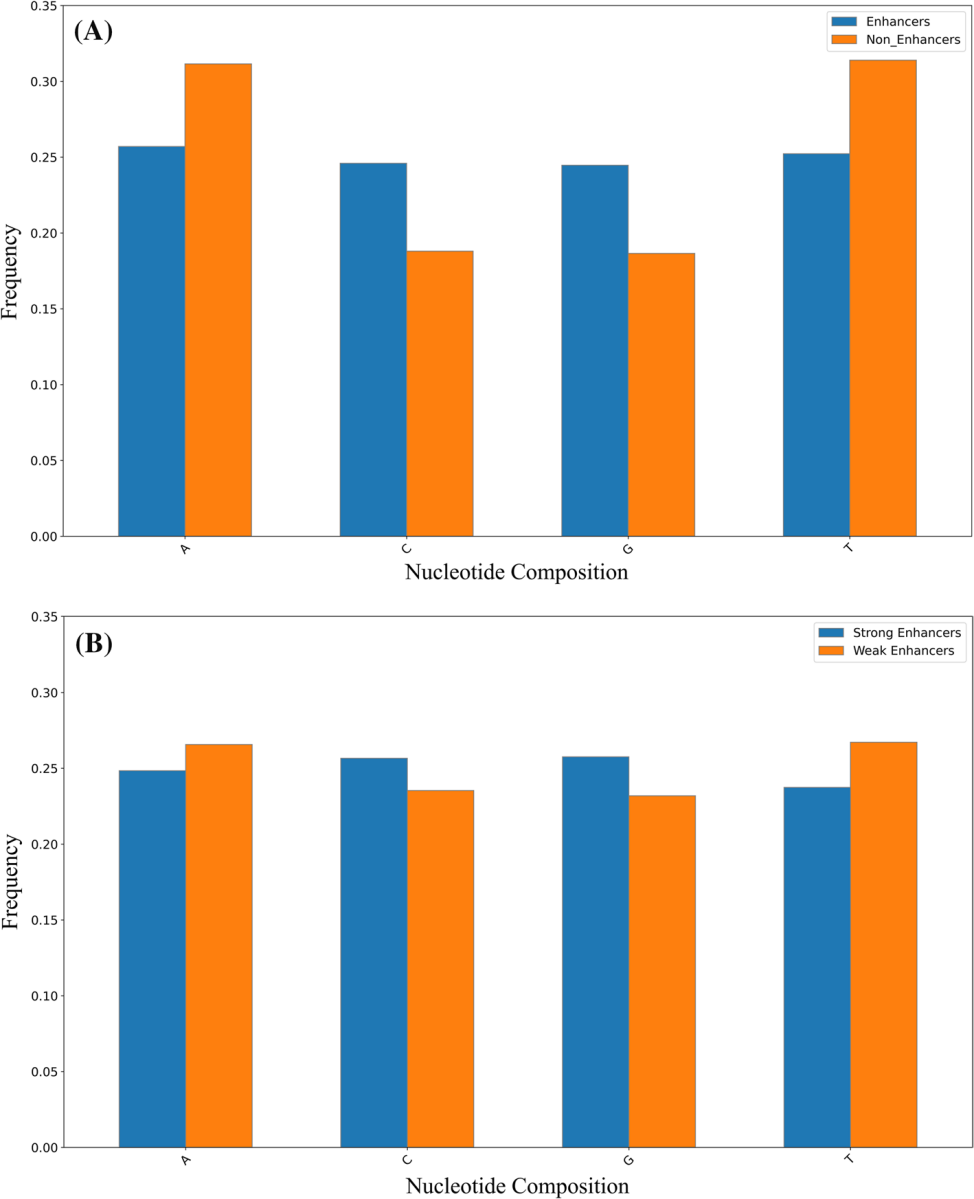
**A** Nucleotide compositions of enhancers and non-enhancers. **B** Nucleotide compositions of strong and weak enhancers

In recent years, nucleotide compositions of DNA sequences have been widely used to identify functional elements [40, 41]. In order to display the distribution of nucleotide of enhancer sequence intuitively. Figure 2 shows the difference in nucleotide compositions between enhancers and non-enhancers and between strong enhancers and weak enhancers, respectively. As shown in Fig. 2A, the four bases are distributed evenly in the sequence of the enhancers, while non-enhancers accumulate adenine (A) and thymine (T). Non-enhancers contain more than 30% adenine and thymine, and less than 20% cytosine(C) and guanine(G). As shown in Fig. 2B, the strong enhancers are rich in more C, G than A, T, while the weak enhancers have the opposite trend, rich in more A, T. These results indicate that there are differences in nucleotide compositions between enhancers and non-enhancers, and between strong enhancers and weak enhancers, which helps us build models to distinguish them.

### Parameter optimization

We set the upper limit of the training period as 90 epochs, and monitored the change of accuracy on the validation set during training. When the accuracy on the validation set reached a relatively high value and stopped improving in the following 20 Epochs, the training was terminated, and the weight with the highest accuracy on the validation set was saved as the test. Table 1 shows the parameter selection we set in the experiment.

**Table 1:** Hyper-parameters optimization

Hyper-Parameters	Range	Recommendation
Convolutional layer number	[1, 2, 3, 4]	2
Convolutional neurons number	[16, 32, 64, 128, 256]	128,256
Convolutional kernel size	[3, 6, 8, 16, 20, 30]	8
Max Pooling layer size	[2, 4, 6, 8]	2
Dropout rates	[0.1, 0.2, 0.3, 0.5]	0.2
Number of neurons in Bi-LSTM	[16, 32, 50, 64]	64
Optimizer	[SGD, Adam]	Adam
Learning rate	[2e−6, 5e−6, 8e−6, 2e−5]	5e−6, 2e−6
Batch Size	[16, 32, 64, 128]	32

In sequence representation, the model is based on k-mer method to embed words by connecting DNA2vec. In previous studies, different k values were selected for different model frames [42]. In order to test the effect of different values of k-mers, we conducted numerical experiments with k ranging from 3 to 8. As shown in Table 2, when k is 7, the model has better performance in general.

**Table 2:** The results of iEnhancer-DCLA with different values of k-mers on two layers

Stages	k-mers	Acc (%)	Sn (%)	Sp (%)	MCC
First layer	3	76.00	73.00	**79.00**	0.5209
	4	75.25	80.50	70.00	0.5078
	5	76.25	77.50	75.00	0.5252
	6	74.50	**83.50**	65.50	0.4981
	7	**78.25**	78.00	78.50	**0.5650**
	8	75.75	71.00	80.50	0.5173
Second layer	3	69.50	81.00	58.00	0.4007
	4	73.00	**96.00**	50.00	0.5181
	5	76.50	95.00	58.00	**0.5705**
	6	76.00	85.00	67.00	0.5286
	7	**78.00**	87.00	**69.00**	0.5693
	8	74.00	92.00	56.00	0.5145

The highest value achieved on each metric is marked in bold

### The effect of different encoding methods

**Fig. 3:**
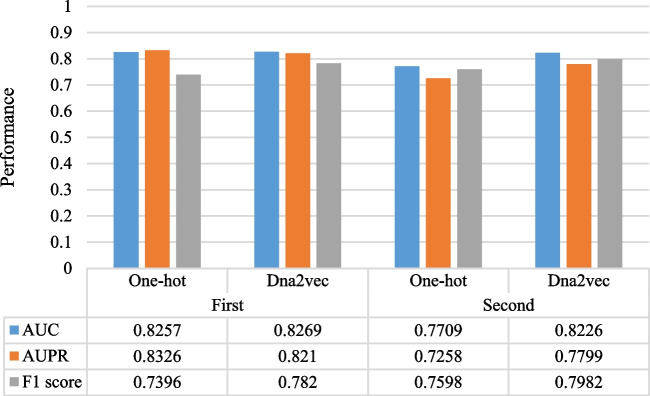
A Comparison of AUC, AUPR and F1 scores at two layers using different encoding schemes

In the past process of DNA sequence processing, one-hot coding has been widely used in various models [23]. One-hot encoding is to encode four bases into four binary numbers, corresponding to each nucleotide has three values set as 0, the other sets as 1. However, if one-hot encoding is carried out for each word, the dimension of the vocabulary will be very large and there will be great sparsity, which will increase the calculation cost. In this paper, we compare the performance of word embedding encoding and one-hot encoding. As shown in Table 3, dna2vec performs better than one-hot encoding at both layers. In Fig. 3, we compare the AUC, AUPR and F1 score of the two encoding methods, it shows that dna2vec has a better performance than one-hot encoding in most of the evaluation indicators for the identification of enhancers and their strength.

**Table 3:** A comparison of two layers using two different encoding schemes

Stages	Encoding	Acc(%)	Sn(%)	Sp(%)	MCC
First layer	One-hot	75.00	71.00	**79.00**	0.5016
	Dna2vec	**78.25**	**78.00**	78.50	**0.5650**
Second layer	One-hot	72.50	87.00	58.00	0.4702
	Dna2vec	**78.00**	**87.00**	**69.00**	**0.5693**

The highest value achieved on each metric is marked in bold

### Discussion on effects of each module of the model

We also discuss the influence of each module of A on the classification effect. In order to select the best model, we constructed four deep learning models, including CNN, BiLSTM, CNN combined with BiLSTM, CNN combined with BiLSTM and attention mechanism. Tables 4 and 5 list the classification performance of the different models. CNN-BiLSTM-Attention achieves the best performance in two stages. In addition, the experiments also show that the higher-order features selected after the attention mechanism are beneficial to improve the prediction ability of the model.

**Fig. 4:**
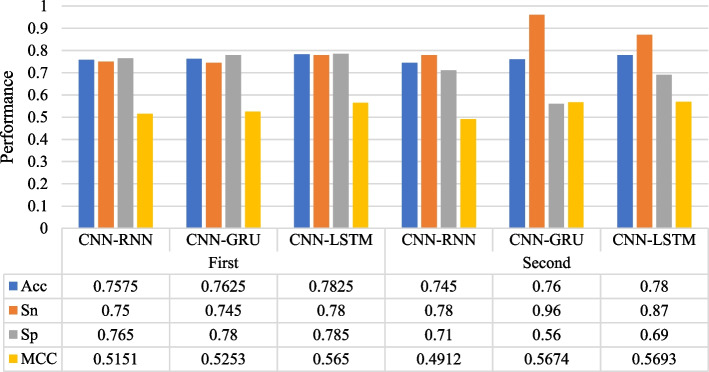
A Comparison of the influence of three different sequence correlation information extraction structures on our model (Bi-RNN, Bi-GRU, Bi-LSTM).

Recurrent neural network is a kind of recursive neural network which takes sequence data as input and carries on recursion in the evolution direction of sequence and all nodes are linked by chain. Gate Recurrent Unit (GRU) is a Recurrent Neural Network (RNN). Compared with LSTM, there are only two gates in GRU model: update gate and reset gate. Simple RNN has no long-term memory, GRU and LSTM can avoid the problem of gradient disappearance. We compare the performance of CNN + RNN, CNN + GRU and CNN + LSTM for the long - term dependence information of extracted sequence. As shown in Fig. 4, CNN + LSTM brings better predictive performance to the model at both stages. We believe that CNN + LSTM solves the problems of gradient disappearance and gradient explosion in the training process of long sequences and can perform better in long sequences.

**Table 4:** Performance comparison of various deep learning models on identifying enhancers

Stages	Model	Acc(%)	Sn(%)	Sp(%)	MCC
First layer	CNN	75.00	79.00	71.00	0.5016
	BiLSTM	74.00	82.50	65.50	0.4871
	CNN-BiLSTM	76.75	78.00	75.50	0.5352
	CNN-BiLSTM-Attention	78.25	78.00	78.50	0.5650

**Table 5:** Performance comparison of various deep learning models on identifying enhancers strength

Stages	Model	Acc(%)	Sn(%)	Sp(%)	MCC
First layer	CNN	70.50	92.00	49.00	0.4541
	BiLSTM	73.00	89.00	57.00	0.4855
	CNN-BiLSTM	75.50	92.00	59.00	0.4855
	CNN-BiLSTM-Attention	78.00	87.00	69.00	0.5693

### Model interpretation

**Fig. 5:**
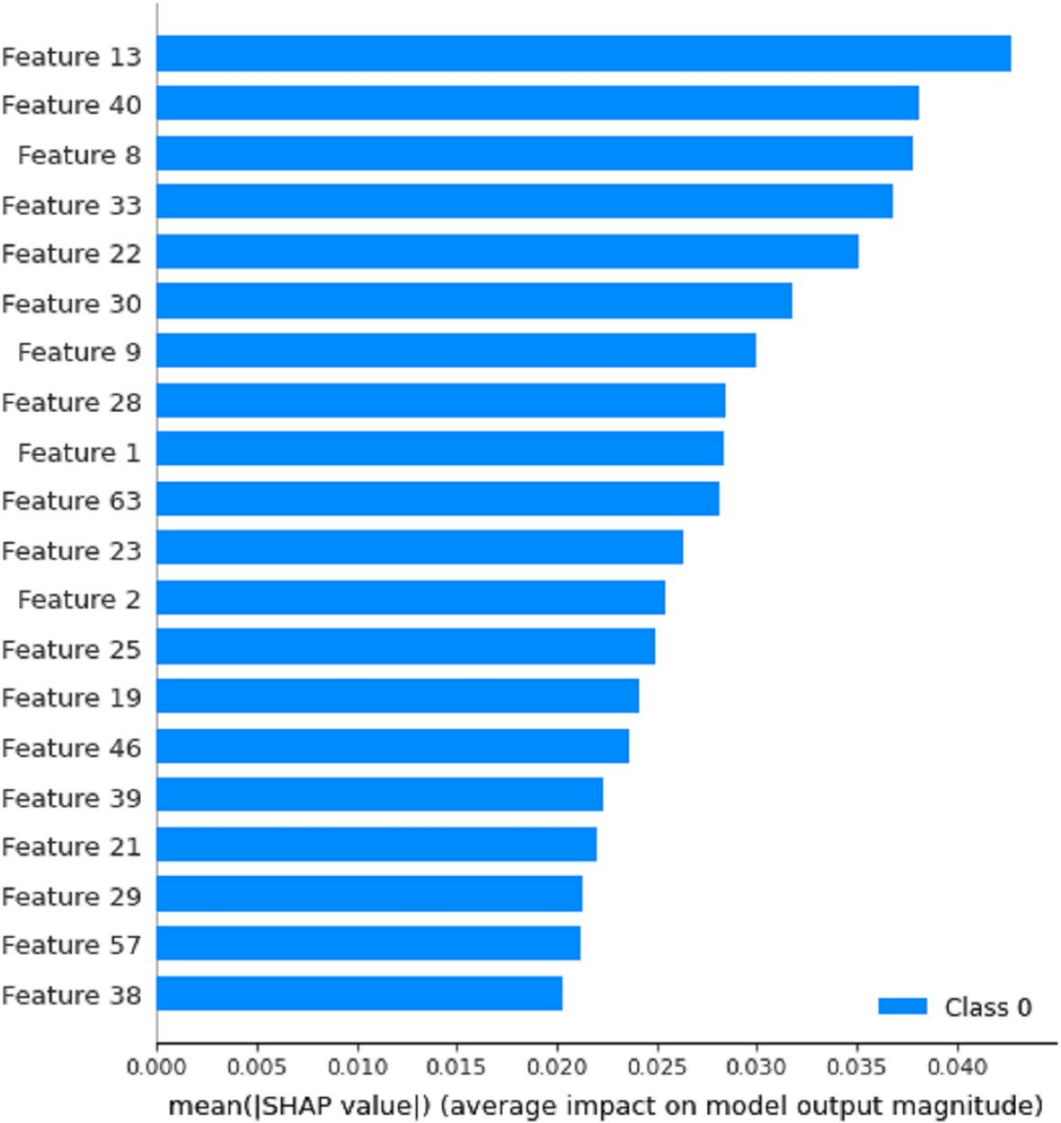
The 20 most influential features of iEnhancer-DCLA.

**Fig. 6:**
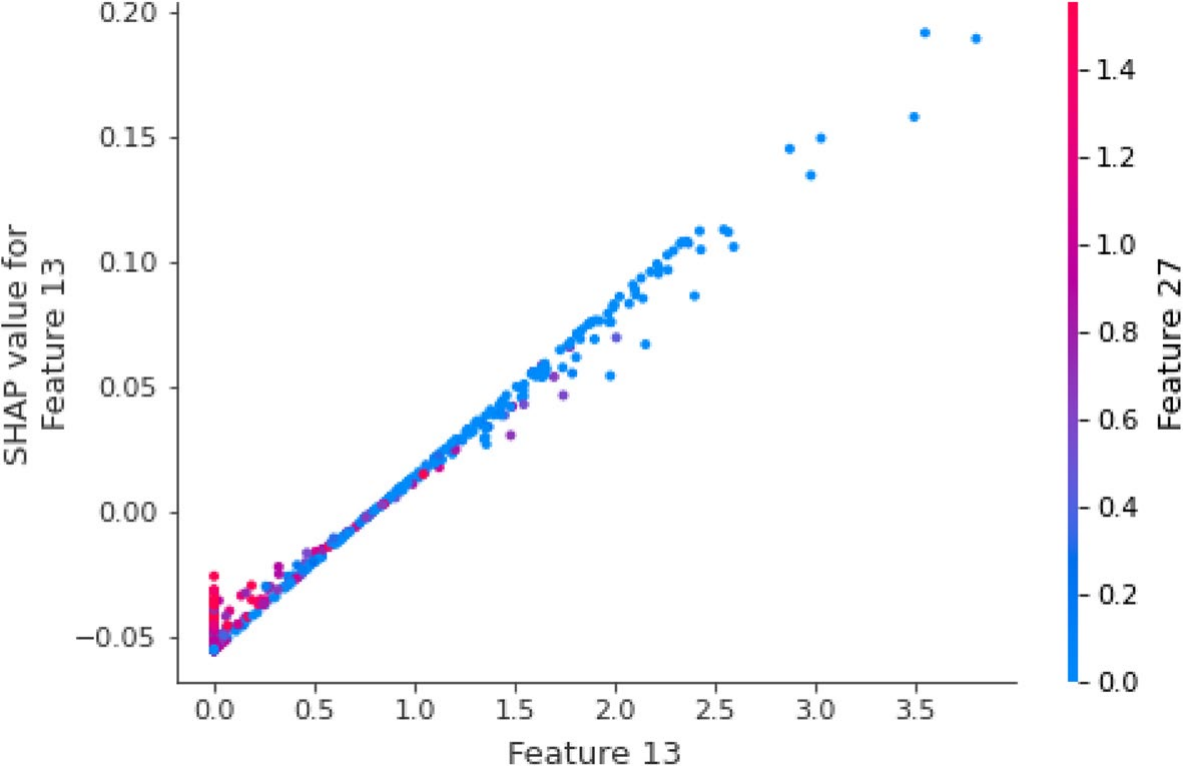
The interaction between features obtained by iEnhancer-DCLA at the attention layer

Many models established by deep learning methods lack interpretability. For us, the model is like a black box, and we only need to design the network structure and relevant parameters to get the results. In recent years, the interpretability of models has gradually become an important research direction of machine learning and deep learning. For example, the ‘SHAP’ method proposed in 2017 can be used to explain various models [26]. We use the SHAP method to explain the interaction between features and the eventual impact of each feature on model classification. We use UMAP [43] dimensionality reduction visualization technology to map the embedding layer and attention mechanism layer of iEnhancer-DCLA into two-dimensional space for feature representation in Additional file 1: Fig. S2. After passing through the attention mechanism layer, the data has an obvious tendency to cluster into two categories, which can be considered that the model has extracted effective sequence features. We pass the model through the feature vector behind the attention layer to the SHAP method. The Fig. 5 shows the sum and average shapley values of all features of all samples, which can reflect the importance of features. It can be seen that the extracted feature 13 has the most significant influence on the final effect of the model. To understand how a single feature affects the output of the model, feature 13 is compared with other sample features. As shown in Fig. 6, red represents feature 27 with a higher shapley value and blue represents a lower value. When feature 13 has a smaller shapley value, feature 27 has a higher value, and when feature 13 has a higher value, feature 27 brings a lower shapley value. The feature coloring of feature 13 shows that it has a negative correlation with feature 27.

### Model evaluation

In this paper, we adopt 5-fold cross-validation to select the best weight. We randomly divided the training dataset into five equal but disjoint subsets. In each fold, we used one of them as the validation set and four as the training set. This process is repeated until all subsets have been validated once. Tables 6 and 7 show the results of 5-fold cross-validation on the benchmark data set at two stages, respectively, to test the learning efficiency and stability of the model. Overall, according to four different evaluation metrics for evaluation, the performance of iEnhancer-DCLA remains consistent across 5-folds.

**Table 6:** The cross-validation results achieved by the iEnhancer-DCLA on identifying enhancers

Stages	Tra : Val (4:1)	Acc(%)	Sn(%)	Sp(%)	MCC
First layer	1	86.83	88.34	85.31	0.7369
	2	84.54	84.57	84.50	0.6907
	3	81.91	85.11	78.71	0.6395
	4	80.73	81.06	80.39	0.6146
	5	82.61	81.81	83.42	0.6524
	Mean	83.32	84.18	82.45	0.6668

**Table 7:** The cross-validation results achieved by the iEnhancer-DCLA on identifying enhancers strength

Stages	Tra : Val (4:1)	Acc(%)	Sn(%)	Sp(%)	MCC
Second layer	1	84.16	86.39	81.94	0.6840
	2	83.36	92.86	73.85	0.6795
	3	82.35	83.96	80.73	0.6472
	4	83.09	87.06	79.11	0.6638
	5	83.56	96.09	71.02	0.6933
	Mean	83.30	89.27	77.33	0.6736

### Performance comparison with existing methods

**Fig. 7:**
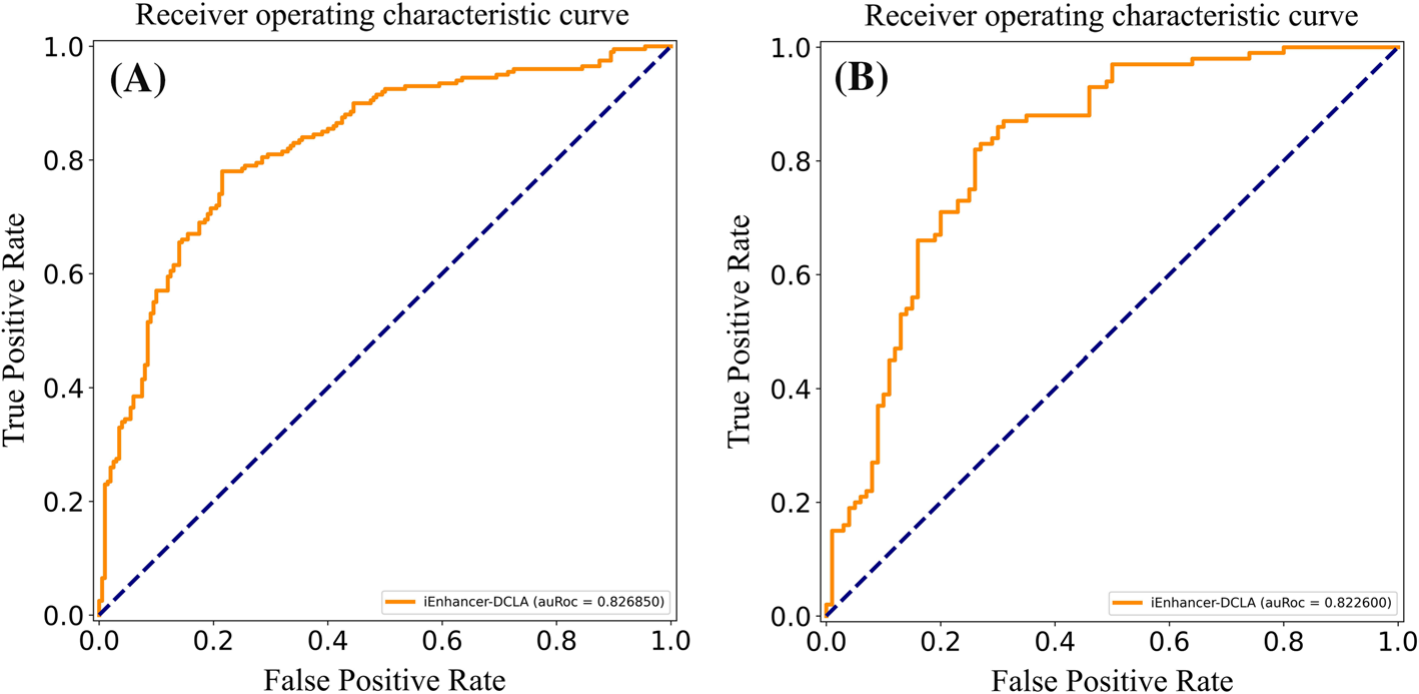
The ROC curve for classifying in the independent test datasets: **A** Layer 1: (Identify Enhancers) **B** Layer 2: (Identify Enhancers’ Strength)

The identification of enhancers and their strength is a complex and important problem. Generally speaking, training different datasets will get different prediction results. In order to objectively evaluate the prediction performance of our model, we select the same evaluation indicators. These methods and our method use the same dataset training and independent test dataset testing. As shown in Table 8, in the first layer of the independent test dataset, our model is slightly lower than iEnhancer-ECNN in the thousandths of Sn, but superior to other methods. Sp is only lower than that of iEnhancer-EBLSTM, which is better than other models. iEnhancer -DCLA is better than other models in ACC, and MCC. In the second layer of the independent test dataset, our method is only below iEnhancer-2 L in Sp. There is a certain deviation between Sn and Sp in our model. In practical application, we prefer to confirm that they are real enhancers, so a higher Sn is acceptable. In the other three evaluation parameters, ACC and Sn values of our model increased by more than 10% and 6% respectively, and MCC increased by more than 0.2. We also retrieved the AUC values of some models for comparison. The AUC values of iEnhancer-2 L, EnhancerPred and iEnhancer-EL were 0.8062, 0.8013 and 0.8173 in the first layer, respectively. As shown in Fig. 7A, the AUC value of our model is 0.8269, which is superior to the above model. In the second layer, the AUC values of the above models for comparison are 0.6678, 0.5790, 0.6801 respectively. As shown in Fig.7B, the AUC value of iEnhancer-DCLA was 0.8226, an increase of 0.14. In summary, our proposed the iEnhancer-DCLA shows the best performance in most evaluation parameters, and can learn the features of enhancer sequences well and make good predictions.

**Table 8:** Identifying enhancers (First layer) and their strengths (Second layer) in the independent test datasets compared to other existing methods

Stages	Method	Acc(%)	Sn(%)	Sp(%)	MCC
First layer	iEnhancer-2L	73.00	75.00	71.00	0.4604
	EnhancerPred	74.00	73.50	74.50	0.4800
	iEnhancer-EL	74.75	71.00	78.50	0.4964
	iEnhancer-ECNN	76.90	**78.50**	75.20	0.5370
	iEnhancer-XG	75.75	74.00	77.50	0.5150
	iEnhancer-EBLSTM	77.20	75.50	**79.50**	0.5340
	iEnhancer-DCLA	**78.25**	78.00	78.50	**0.5650**
Second layer	iEnhancer-2L	60.50	47.00	**74.00**	0.2181
	EnhancerPred	55.00	45.00	65.00	0.1021
	iEnhancer-EL	61.00	54.00	68.00	0.2222
	iEnhancer-ECNN	67.80	79.10	56.40	0.3680
	iEnhancer-XG	63.50	70.00	57.00	0.2720
	iEnhancer-EBLSTM	65.80	81.20	53.60	0.3240
	iEnhancer-DCLA	**78.00**	**87.00**	69.00	**0.5693**

The highest value achieved on each metric is marked in bold

## Conclusion

Enhancers are DNA sequences that increase promoter activity and thus gene transcription frequency. Identification of enhancers and their strength is of great significance for drug development and synthetic biology. In this study, we developed a new deep learning model called iEnhancer-DCLA. This model firstly combines word embedding and k-mer analysis as sequence encoding methods, and then uses CNN, Bi-LSTM and attention mechanism to extract features and complete classification tasks. We use cross-validation to select the best weights for testing. The experimental results show that word embedding can express DNA sequences well, and the proposed model performs better than other existing advanced models using the same benchmark dataset in identifying enhancers and predicting their strength. In addition, in order to further improve the prediction effect of the model, our subsequent work is mainly focused on exploring sequence coding schemes, feature extraction methods and data augmentation.

## Supplementary Information

**Additional file 1: Fig. S1.** Dimension changes of iEnhaner-DCLA under eachmodule. **Fig. S2.** Two-dimensional feature representation ofenhancers and non-enhancers’ data before and after model training.

## Data Availability

All the raw data are available at http://bioinformatics.hitsz.edu.cn/iEnhancer-2 L/ (Liu et al.,2015), and all code scripts used are available at https://github.com/WamesM/i. Enhancer-DCLA.
