# Supplementary material for: iEnhancer-DCLA: using the original sequence to identify enhancers and their strength based on a deep learning framework
Source: BMC Bioinformatics. 2022 Nov 14;23:480. doi: 10.1186/s12859-022-05033-x (PMC9664816; doi:10.1186/s12859-022-05033-x)
Supplement: Supplementary file 1 — Additional file 1: Fig. S1. Dimension changes of iEnhaner-DCLA under eachmodule. Fig. S2. Two-dimensional feature representation ofenhancers and non-enhancers’ data before and after model training. [file 12859_2022_5033_MOESM1_ESM.docx]

The detailed structure of the iEnhancer-DCLA model is presented in Figure S1. The sequence length we input is 200bp. Sequence is represented as 200*100-D matrix by embedding layer, and transformed into 50*128-D matrix by sequential (CNN) and bidirectional LSTM modules, then transformed into 128-D feature vectors by attention mechanism module, and finally into the fully connected layers for prediction.


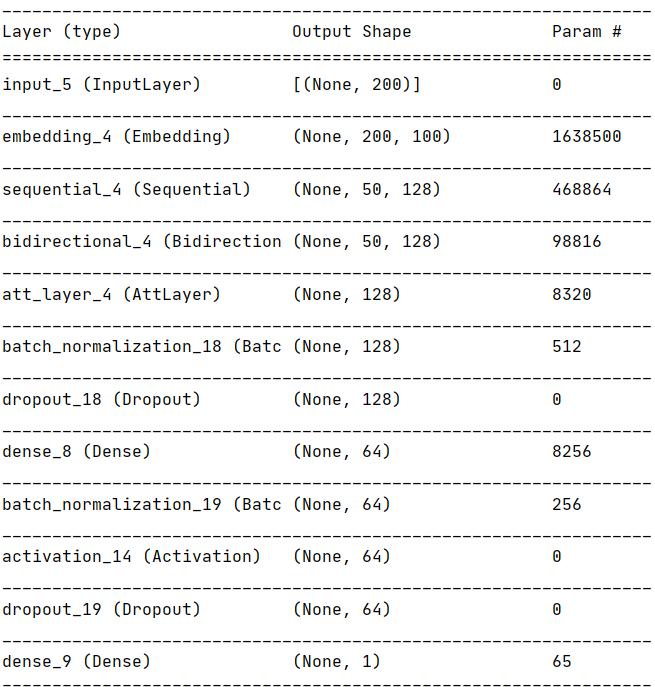


Figure S1. Dimension changes of iEnhaner-DCLA under each module.

UMAP (Uniform Manifold Approximation and Projection for Dimension Reduction) is a dimension reduction technique. As shown in Figure S2, the left figure shows the two-dimensional feature representation of the sequence at the embedding layer. The right figure shows the feature representation after the action of the attention mechanism.

| (A)  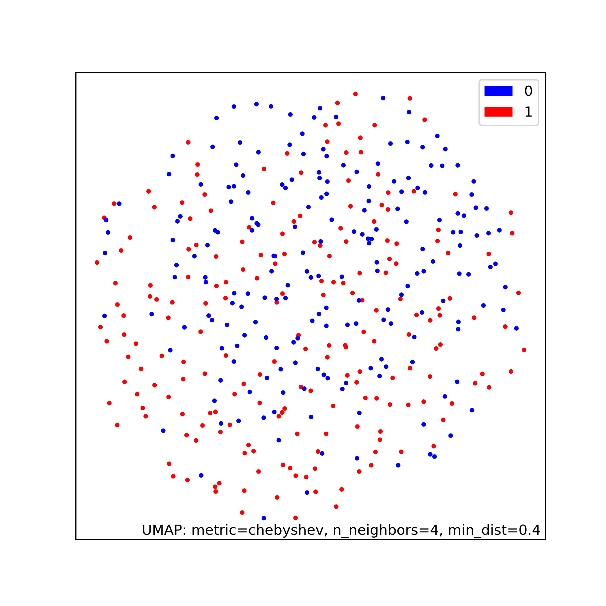 | (B)  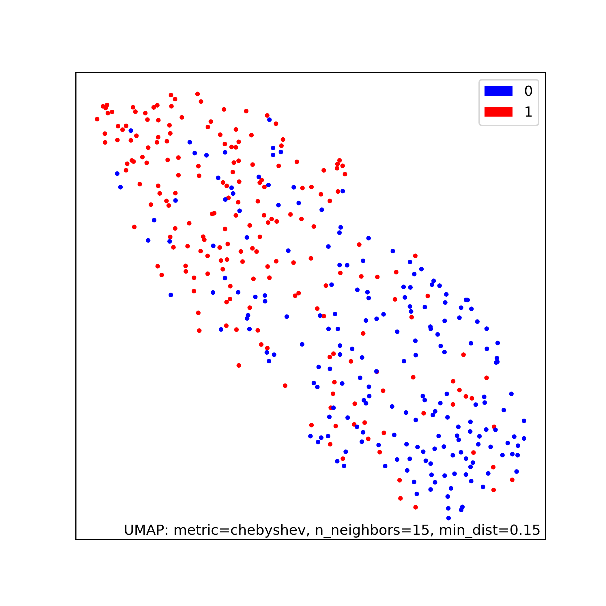 |
| --- | --- |

Figure S2. Two-dimensional feature representation of enhancers and non-enhancers’ data before and after model training.
